# Supplementary material for: From sequence to enzyme mechanism using multi-label machine learning
Source: BMC Bioinformatics. 2014 May 19;15:150. doi: 10.1186/1471-2105-15-150 (PMC4229970; doi:10.1186/1471-2105-15-150)
Supplement: Additional file 2 — Java code of ml2db. Additional file ml2db_code.tar.gz contains the Java source code to run the multi-label machine learning experiments and save the results to database. The code’s Javadoc is included. [file 1471-2105-15-150-S2.zip › additional file 2/ml2db/utils/doc/uk/ac/standrews/utils/main/NumberUtils.html]

NumberUtils


JavaScript is disabled on your browser.


- Overview
- Package
- Class
- Use
- Tree
- Deprecated
- Index
- Help

- Prev Class
- Next Class

- Frames
- No Frames

- All Classes

- Summary:
- Nested |
- Field |
- Constr |
- Method

- Detail:
- Field |
- Constr |
- Method


uk.ac.standrews.utils.main

## Class NumberUtils

- java.lang.Object
- - uk.ac.standrews.utils.main.NumberUtils

- ---

    

  ```
  public class NumberUtils
  extends java.lang.Object
  ```

  Class

  Version:
  :   22 Jan 2009

  Author:
  :   Luna De Ferrari luna.deferrari-at-ed.ac.uk

- - ### Constructor Summary

    Constructors

    Constructor and Description

    `NumberUtils()`
  - ### Method Summary

    Methods

    | Modifier and Type | Method and Description |
    | --- | --- |
    | `static java.lang.String` | `doubleTo2DecimalsString(java.lang.Double aDouble)` Get a percentage representation of a double |
    | `static java.lang.String` | `formatPercentOneFraction(double num)` Takes a double with any number of decimals and returns a String representation of a percentage 0.85842 -> 85.8% |
    | `static java.lang.String` | `getValue(java.lang.Double value)` Gets a string value usable for SQL DOUBLE column. |
    | `static boolean` | `isMultipleOf(int multiple, int divisor)` checks if a number is a multiple of another number. |
    | `static java.lang.Double` | `roundDouble(int digits, java.lang.Double value)` From http://stackoverflow.com/questions/153724/how-to-round-a-number-to-n-decimal-places-in-java |

    - ### Methods inherited from class java.lang.Object

      `equals, getClass, hashCode, notify, notifyAll, toString, wait, wait, wait`

- - ### Constructor Detail


    - #### NumberUtils

      ```
      public NumberUtils()
      ```
  - ### Method Detail


    - #### doubleTo2DecimalsString

      ```
      public static java.lang.String doubleTo2DecimalsString(java.lang.Double aDouble)
      ```

      Get a percentage representation of a double

      Parameters:
      :   `fraction` - e.g. 0.75

      Returns:
      :   a percentage, e.g. 75%
          public static String getPercentage(Double fraction){ Double
          percent = new Double(fraction); NumberFormat percentFormatter =
          NumberFormat.getPercentInstance(Locale.getDefault()); return
          percentFormatter.format(percent);
          }


    - #### formatPercentOneFraction

      ```
      public static java.lang.String formatPercentOneFraction(double num)
      ```

      Takes a double with any number of decimals and returns a String
      representation of a percentage 0.85842 -> 85.8%


    - #### getValue

      ```
      public static java.lang.String getValue(java.lang.Double value)
      ```

      Gets a string value usable for SQL DOUBLE column. The string must be
      either double precision numeric or null: NaN or +/-inf are not acceptable
      values.

      Parameters:
      :   `the` - value

      Returns:
      :   the double precision value string or null if = NaN or = +/-inf


    - #### isMultipleOf

      ```
      public static boolean isMultipleOf(int multiple,
                         int divisor)
      ```

      checks if a number is a multiple of another number. 0 always returns
      true.

      Parameters:
      :   `step` -
      :   `i` -

      Returns:


    - #### roundDouble

      ```
      public static java.lang.Double roundDouble(int digits,
                                 java.lang.Double value)
      ```

      From http://stackoverflow.com/questions/153724/how-to-round-a-number-to-n-decimal-places-in-java

      Parameters:
      :   `digits` - the desired number of digits
      :   `value` - a double

      Returns:
      :   a rounded double


- Overview
- Package
- Class
- Use
- Tree
- Deprecated
- Index
- Help

- Prev Class
- Next Class

- Frames
- No Frames

- All Classes

- Summary:
- Nested |
- Field |
- Constr |
- Method

- Detail:
- Field |
- Constr |
- Method
